# Exogenous estrogen therapy, testicular cancer, and the male to female transgender population: a case report

**DOI:** 10.1186/s13256-018-1894-6

**Published:** 2018-12-19

**Authors:** Gursimran Chandhoke, Bobby Shayegan, Sebastien J. Hotte

**Affiliations:** 1 Department of Oncology, McMaster University, Juravinski Cancer Centre, 699 Concession St, Hamilton, ON L8V 5C2 Canada; 2 Department of Urology, McMaster University, St. Joseph’s Healthcare, 50 Charlton Ave E, Hamilton, ON L8N 4A6 Canada

**Keywords:** Testicular cancer, Testicular dysgenesis syndrome, Transgender

## Abstract

**Background:**

Over the last 40 years, there has been a significant increase in the incidence of testicular cancer. The epidemiologic evidence to understand this phenomenon is unclear, however exogenous estrogen exposure is thought to be a driver in the development of testicular cancer. This is of particular importance in the transgender population because utilization of exogenous estrogen therapy is an essential aspect of the transition process.

**Case:**

We present the case of a 38-year-old Caucasian male to female transgender patient who presented with metastatic testicular cancer 15 months after initiating estrogen therapy. She presented to our emergency department with worsening back pain and fatigue. A clinical examination revealed a right-sided testicular mass. A computed tomography scan of her abdomen/pelvis identified a right groin lesion measuring 6.4 cm, a retroperitoneal mass causing right-sided hydronephrosis, an extensive deep vein thrombosis, and pathologic abdominal lymphadenopathy. Germ cell tumor markers revealed an alpha-fetoprotein of < 2.5 μg/L and a beta-human chorionic gonadotrophin of 2526 IU/L. Her lactate dehydrogenase was 5294 U/L. Medical oncology advised the discontinuation of hormonal therapy at this time. On the basis of elevation in germ cell tumor markers and the burden of disease, she was treated with four cycles of bleomycin, etoposide, and cisplatin chemotherapy. A decision to defer upfront radical inguinal orchiectomy was made due to not wanting to have an early interruption in anticoagulation.

Following the completion of the chemotherapy, a 6 cm retroperitoneal mass persisted. Due to the location of the mass and surgical morbidity associated with excision, she was followed with positron emission tomography-computed tomography by Uro-oncology, with no evidence of recurrent disease 2 years since the time of diagnosis.

**Conclusions:**

While there are recognized risks associated with estrogen therapy less is known about the extent to which exogenous estrogen can serve as a driver of malignancy. With recent experimental evidence revealing a pro-growth impact of estrogen on human testicular cells, continued reporting of similar cases in the literature is imperative to see if a link between exogenous estrogen exposure and testicular cancer exists.

## Background

Over the last 40 years, there has been a significant increase in the incidence of testicular cancer (TC). While the epidemiologic evidence to understand this phenomenon is unclear, testicular dysgenesis syndrome (TDS) is a theory that attributes the increased rate of disorders of male fertility (impaired spermatogenesis, cryptorchidism, and TC) to a common error in gonocyte development that is in part driven by exposure to excess estrogen.

This is of particular importance in the male to female transgender population, because utilization of sustained exogenous estrogen therapy is an essential aspect of the transition process. While there are recognized risks associated with estrogen therapy, (for example, thrombosis) less is known about the extent to which exogenous estrogen can serve as a driver of malignancy such as TC.

Here we discuss the case of a male to female transgender patient who developed TC after many months of exogenous estrogen therapy, review the biologic plausibility, and review the literature for similar cases for which there is only one case. Continued reporting of similar cases in the literature is imperative to see if a link between exogenous estrogen exposure and TC exists.

## Case presentation

A previously healthy 38-year-old Caucasian male to female transgender patient was placed on hormonal therapy for 15 months with estradiol 2 spironolactone while awaiting gender re-assignment surgery. There was no other previous past medical or surgical history. She was not taking any other medications and had a non-anaphylactic allergy to penicillins. She is married with two children and is employed as a truck driver. She never uses recreational drugs, has an infrequent alcohol intake (< 1/week), and is an ex-tobacco smoker. There was no family history of TC.

**Fig. 1:**
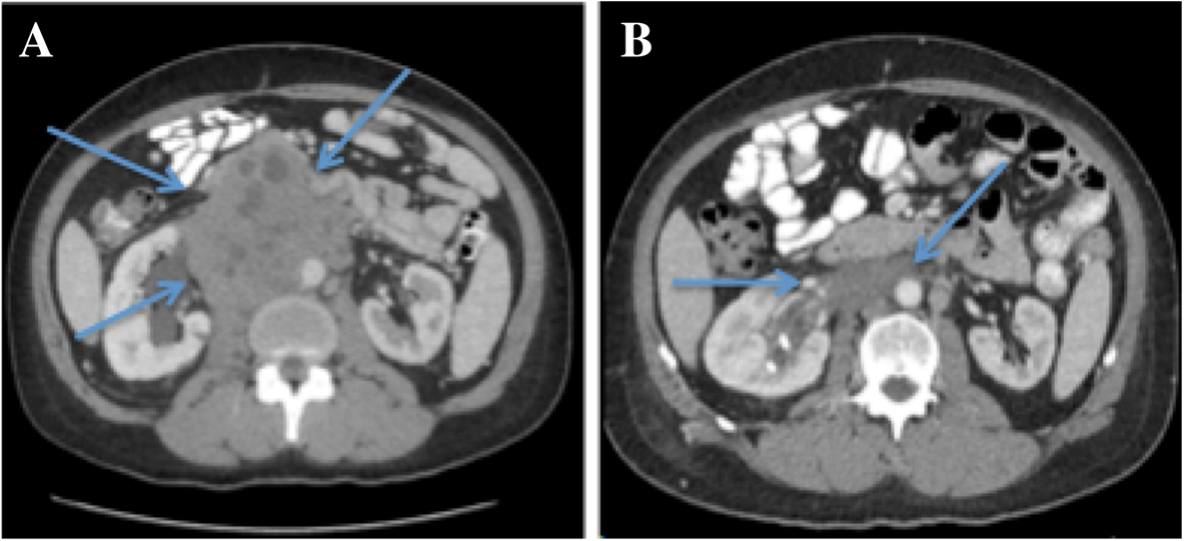
**a** Pre-treatment computed tomography scan of the abdomen revealing a large, 11 cm retroperitoneal mass causing right-sided hydronephrosis. **b** Post-treatment (four cycles of bleomycin, etoposide, and cisplatin) computed tomography scan of the abdomen showing significant interval decrease in the retroperitoneal mass. Arrows are pointing to the retroperitoneal mass. Pre and post treatment

In early 2016, she developed progressive right-sided scrotal swelling, abdominal and back pain, fatigue, and weight loss of 7 kg. With ongoing pain, she presented to our Emergency Department in June 2016. On presentation her vital signs were 36.1 °C, heart rate (HR) 99 beats per minute, blood pressure (BP) 169/97, and 99% on room air with 18 respirations a minute. A clinical examination revealed no focal neurological deficits, normal cardiorespiratory examinations, palpable adenopathy in the left supraclavicular fossa but no palpable axillary or inguinal adenopathy. Her abdomen was soft and non-tender, there was no organomegaly, and a genitourinary examination revealed a large right-sided testicular mass. A computed tomography (CT) scan of her abdomen/pelvis identified a right groin lesion measuring 6.4 × 4.9 × 6.3 cm, and a retroperitoneal mass (11.5 × 10.6 × 17.4 cm) causing right-sided hydronephrosis (see Fig. 1a). Furthermore, an extensive deep vein thrombosis (DVT) involved the common iliac and femoral vessels. Lastly, there were pathologically enlarged lymph nodes in the celiac axis, retrocrural space, and external iliac chains. A CT scan of her thorax revealed left-side neck adenopathy measuring 3 × 3 cm but no parenchymal lung lesions were noted. Laboratory investigations revealed: hemoglobin 103 g/l, white blood cells (WBC) 10.9 × 10^9/^L (absolute neutrophil count of 8.9), platelets of 446 × 10^9/^L, sodium 130 mmol/L, potassium 4.2 mmol/l, creatinine 127 umol/L, with remaining extended electrolytes (Ca, Mg) and liver panel within normal limits. At the time of diagnosis, lactate dehydrogenase (LDH) was 5294 U/L and the tumor markers alpha-fetoprotein (AFP) and beta-human chorionic gonadotrophin (β-HCG) were < 2.5 μg/L and 2526 IU/L, respectively. Overall her clinical stage was IIIC (Tx,N3,M1a,S3).

She was seen by the Thrombosis and Interventional Radiology services; she was started on dalteparin for anticoagulation, and a nephrostomy tube was inserted on the right side.

While it would be standard practice to pursue a radical orchiectomy prior to the initiation of chemotherapy this was deferred in this case for a variety of reasons. First, it was felt that an early initiation of chemotherapy was indicated to reduce the high burden of disease as she was highly symptomatic and unstable. Second, given the extensive clot burden, there were concerns with the early interruption of anticoagulation for a surgical procedure. Last, although the left-sided neck disease could have been biopsied for diagnosis, it was felt to be too risky given its location relative to vascular structures.

Immediately following this, Medical Oncology initiated treatment with chemotherapy using bleomycin, etoposide, and cisplatin (BEP) and advised our patient to discontinue hormonal therapy.

Given the overall burden of disease, a decision was made to pursue four cycles of chemotherapy with BEP which was given between June 2016 and October 2016. Tumor markers following the completion of treatment were AFP 2.7 μg/L, β-HCG 2.4 IU/L, and LDH 247 U/L.

She then underwent a radical right inguinal orchiectomy in October 2016 (preoperatively she received 2 grams of cefazolin intravenously) with pathology revealing scarring related to treatment effect but no evidence of malignancy. Re-staging CT scans revealed a residual retroperitoneal mass measuring 6.1 cm × 2.4 cm and persistent thrombosis of the inferior vena cava (IVC; Fig 1b).

With a residual mass, she was referred to Uro-oncology in November 2016 for consideration of a retroperitoneal lymph node dissection. Ultimately, the decision was made to follow this residual mass with serial positron emission tomography (PET) and CT scans. The basis for this was twofold. First, the elevated β-HCG, the normal AFP, and overall clinical picture suggested that this was a seminoma, and second, the location of the lesion would result in a surgical risk and morbidity that would outweigh any potential benefit. Since then routine scans have been completed every 3 months with no evidence of disease recurrence.

In November 2016, following the completion of chemotherapy, because of the persistence of thrombosis on imaging, and our patient’s wish to restart therapy with estrogen (this was based on her desire to continue with the transition from male to female) she was switched from dalteparin to rivaroxaban 20 mg orally once a day. She was counseled on the risk of thrombosis with the resumption of exogenous estrogen therapy, and there was a discussion surrounding the experimental evidence suggesting a possible link between estrogens and TC.

As of June 2018, she has shown no evidence of recurrence; her AFP and β-HCG remain within normal limits. She undertook orchiectomy of the remaining testicle in August 2017, which showed no evidence of malignancy or other significant pathology. She continues with indefinite anticoagulation given her ongoing use of exogenous estrogen.

## Case summary

In summary, we have presented a case of a previously healthy 38-year-old male to female transgendered patient who, after being on exogenous estrogen therapy for 15 months, presented with a stage IIIC germ cell tumor. She was treated with BEP × four cycles, after which she underwent a radical inguinal orchiectomy which revealed no evidence of residual malignancy. Restaging CT scans following the completion of chemotherapy revealed a 6 cm retroperitoneal mass which was not amenable to surgical resection. As such, she was followed with serial imaging with PET/CT scans every 3 months. It is now over 2 years since the time of diagnosis, and she has no evidence of disease recurrence.

## Discussion

TC is the most common malignancy in men aged 15–40. Over 95% are germ cell tumors, of which the main classification is seminoma and non-seminoma [1]. Non-seminomas include tumors of the yolk sac, embryonal cell, teratoma, and choriocarcinoma, while pure seminomas have no non-seminomatous components present. Biochemically, pure seminomas do not produce AFP but may produce a small amount of β-HCG.

Patients with TC will often present with a mass or lump in the testis. Signs and symptoms suggestive of loco-regional/metastatic disease include swelling of the lower extremities, back pain, dyspnea, or hemoptysis.

In patients where TC is suspected, baseline investigations should include routine bloodwork with AFP, β-HCG, and LDH. Imaging studies should include a scrotal ultrasound and CT of the chest/abdomen/pelvis (in cases of stage I seminoma, a chest X-ray is suitable). Additional investigations such as a bone scan or CT scan of the head are indicated if there are symptoms suggestive of disease in these locations.

On completion of these investigations, patients should be assessed for consideration of orchiectomy [2]. Orchiectomy is a simple, straightforward procedure that is unlikely to cause a significant delay toward the start of chemotherapy. Given that a pathologic assessment of the testicle provides vital prognostic information and guides treatment decisions (for example, seminoma, non-seminoma, and teratoma), orchiectomy should only be delayed in exceptional circumstances (life-threatening disease, significant overall burden of disease).

In patients with localized disease, tumor markers should decline as predicted by their half-lives (AFP < 7 days, ß-HCG < 3 days).

In the metastatic setting, defining the tumor as seminoma versus non-seminoma, the level of tumor markers, and the sites of metastases helps to stratify risk and define the approach to therapy (see Table 1) [3]. Based on international randomized clinical trial data, seminomas and good prognosis non-seminomas can be treated with three cycles of BEP chemotherapy whereas intermediate/poor risk non-seminomas are most often treated with four cycles of BEP.

**Table 1:** Risk stratification for metastatic seminoma and non-seminoma

Risk stratification	Seminoma	Non-seminoma
Good	Any hCG	AFP < 1000 ng/ml
		hCG < 5000 mU/ml
		LDH < 1.5 × ULN
	Any LDH	Non-pulmonary visceral metastases absent
		Gonadal or retroperitoneal primary tumor
Intermediate	Any hCG	AFP 1000–10,000 ng/ml
	Any LDH	hCG 5000–50,000 mU/ml
		LDH 1.5–10 × ULN
	Any primary site	Non-pulmonary visceral metastases absent
	Non-pulmonary visceral metastases absent	
		Gonadal or retroperitoneal primary tumor
Poor	None	AFP > 10,000 ng/ml
		hCG > 50,000 mU/ml
		LDH > 10 × ULN
		Mediastinal primary site
		Non-pulmonary visceral metastases present

*AFP* alpha fetoprotein, *hCG* human chorionic gonadotrophin, *LDH* lactate dehydrogenase, *ULN* upper limit of normal (adopted from the International Germ Cell Cancer Collaborative Group risk classification for metastatic disease [4])

Following the completion of therapy, patients should be followed with regular physical examinations, tumor markers, and imaging investigations (see Table 2) [4].

**Table 2:** Recommended follow-up for metastatic seminoma and non-seminoma germ cell tumors

	Year 1	Year 2	Year 3–5
Physical examination	Q3 months	Q3 months	Q6 months
Laboratory investigations including tumor markers (AFP, b-HCG, LDH)	Q3 months	Q3 months	Q6 months
CXR	Q3 months	Q3 months	Q6 months
CT abdomen/pelvis	Q6 months	Q6 months	Q12 months
CT chest	Q12 months	Q12 months	Q12 months

*AFP* alpha-fetoprotein, *b-HCG* beta- human chorionic gonadotrophin, *CT* computed tomography, *CXR* chest X-ray, *LDH* lactate dehydrogenase, *Q* every (adopted from the European Association of Urology [5])

In metastatic non-seminomas, residual masses (with normal tumor markers) may remain following chemotherapy. While a “residual mass” has not been defined in terms of size, consensus guidelines do recommend surgical resection of amenable masses > 1 cm. In almost 50% of the cases, these resected masses demonstrated post treatment necrosis while 10% demonstrated viable cancer cells. While there is no reliable way to predict the likely pathology of these residual masses, favorable factors include absence of teratoma in the primary tumor, large shrinkage of the mass with chemotherapy, and residual size < 1 cm [2].

In metastatic seminomas, residual masses are classified as > 3 cm or < 3 cm. For lesions greater than or equal to 3 cm, fluorodeoxyglucose (FDG)-PET should be obtained for further assessment, while for masses < 3 cm, PET scans are optional. If the PET scan is positive, the treatment of choice would be surgical resection. Radiation therapy may also be considered, although the benefit of this would be less clear [2]. In our case, since the lesion was thought to be a seminoma (normal AFP), monitoring with FDG-PET would be in keeping with consensus guidelines.

### Estrogens and TC

Over the course of the last few decades, the overall incidence of TC has increased and has doubled over the last 40 years in many regions in the world [5]. One theory that looks to explain this phenomenon is TDS, which suggests that the increased incidence of impaired spermatogenesis, reproductive tract abnormalities (for example, cryptorchidism), and TC, all share a common etiology related to errors in gonocyte development [6]. The earliest signs of TC can be found during embryogenesis, in which excessive levels of estrogen during early pregnancy can lead to a disturbance in the development of primordial germ cells destined to form spermatogonia. This results in premalignant cells which may become carcinoma *in situ* after birth. These cells remain dormant until there is a change in their local microenvironment, attributable to changes in (1) androgen levels, (2) excessive estrogens, or (3) exogenous hormonal imbalance [7]. These can be manifested as the hormonal changes of puberty, through exogenous hormonal administration, and from exposure to occupational and environmental estrogenic chemicals.

With this in mind, a 1987 study that assessed the histology of testicles in male to female transgendered patients following exposure to exogenous estrogens found atrophy of the seminiferous tubules, cellular hypertrophy, and hyperplasia of the rete testis, and to a lesser extent of the ductuli efferentes, and of the epididymal ductal epithelium [8].

Furthermore, a recent study has demonstrated a pro-growth impact of estrogens on human testicular germ cells, mediated through the activation of an extracellular regulated kinase and protein kinase A [9].

Our review of the literature reveals one other case of a male to female transgendered patient who, after being on estrogen therapy for 22 years, developed TC that required surgery and four cycles of BEP chemotherapy [10].

Continued reporting of similar cases is imperative to understand any potential putative link between exogenous estrogen exposure and the development of TC. While there is a paucity of data to support or refute this suggestion, completing a screening scrotal ultrasound in male to female transgendered patients prior to being placed on estrogen therapy may be useful and is non-invasive and inexpensive. If a patient is planned to undergo prolonged hormonal therapy, regular interval ultrasounds (for example, every 6 to 12 months) could be considered.

## Conclusions

To the best of our knowledge, this is only the second case in the literature whereby a male to female transgender patient developed TC while on exogenous estrogen therapy. While it is not possible to determine if estrogen is exerting a carcinogenic effect in this setting, continued reporting of similar cases is imperative to assess for this possibility.

## References

[1] Giannandrea F, Paoli D, Figa-Talamanca I, Lombardo F, Lenzi A, Gandini L. Effect of endogenous and exogenous hormones on testicular cancer: the epidemiological evidence. Int J Dev Biol. 2013;57:255–63. 10.1387/ijdb.130015fg. 23784836

[2] Wood L, Kollmannsberger C, Jewett M, Chung P, Hotte S, O’Malley M, Sweet J, *et al.* Canadian consensus guidelines for the management of testicular germ cell cancer. Can Urol Assoc J. 2010;4(2):e19–38. 10.5489/cuaj.815 PMC2845668 20368885

[3] [No authors listed]. International Germ Cell Consensus Classification: a prognostic factor-based staging system for metastatic germ cell cancers. International Germ Cell Cancer Collaborative Group. J Clin Oncol. 1997;15(2):594–603. 10.1200/JCO.1997.15.2.594 9053482

[4] Albers P, Albrecht F, Algaba C, Bokemeyer G, Cohn-Cedermark G, Fizazi K, Horwich A. Guidelines on Testicular Cancer. Eur Urol. 2015;68(6):1054–68. 10.1016/j.eururo.2015.07.044. 26297604

[5] Liu S, Semenciw R, Waters C, Wen SW, Mery LS, Mao Y. Clues to the aetiological heterogeneity of testicular seminomas and non-seminomas: time trends and age-period-cohort effects. Int J Epidemiol. 2000;29:826–31. 10.1093/ije/29.5.826 11034964

[6] Skakkebaek NE, Rajpert-De Meysts E, Main KM. Testicular dysgenesis syndrome: an increasingly common developmental disorder with environmental aspects. Hum Reprod. 2001;16:972–8. 10.1093/humrep/16.5.972 11331648

[7] Almstrup K, Sonne SB, Hoei-Hansen CE, Ottesen AM, Nielsen JE, Skakkebaek NE, Leffers H. From embryonic stem cells to testicular germ cell cancer: should we be concerned? Int J Androl. 2006;29:211–8. 10.1111/j.1365-2605.2005.00643.x 16466542

[8] Sapino A, Pagani A, Godano A, Bussolati G. Effects of estrogens on the testis of transsexuals: a pathological and immunocytochemical study. Virchows Arch A Pathol Anat Histopathol. 1987;411(5):409–14. 10.1007/BF00735221 3116755

[9] Bouskine A, Nebout M, Mograbi B, Brucker-Davis F, Roger C, Fenichel P. Estrogens promote human testicular germ cell cancer through a membrane-mediated activation of extracellular regulated kinase and protein kinase A. Endocrinology. 2008;149(2):565–73. 10.1210/en.2007-1318 18039775

[10] Hannoush, ZC, Ayala, A. Estrogen Exposure and Testicular Choriocarcinoma: Case Report of a Male to Female Transgender Patient. Presented at Endocrine Society's 98th Annual Meeting and Expo, April 2016 – Boston. Available from: http://press.endocrine.org/doi/abs/10.1210/endo-meetings.2016.TB.1.FRI-090

